# Bronchiectasis in the setting of aplasia of the epiglottis

**DOI:** 10.1002/rcr2.430

**Published:** 2019-05-01

**Authors:** Puwakdandawe Weerasinghe, Rahul Thomas, Brent Masters, Nitin Kapur

**Affiliations:** ^1^ Department of Respiratory and Sleep Medicine Queensland Children's Hospital Brisbane Queensland Australia

**Keywords:** Aplasia, epiglottis, bronchiectasis, aspiration

## Abstract

Aplasia of the epiglottis is a rare airway abnormality requiring airway and feeding interventions. We report a case of bronchiectasis in the setting of congenital aplasia of the epiglottis, secondary to early‐life aspiration events. Compensatory mechanisms for airway protection likely develop later in life.

## Introduction

The epiglottis has been suggested to provide upper airway stability and protection 1, 2. Abnormalities of the epiglottis can occur, including hypoplasia and aplasia, where the latter is significantly more uncommon, and the exact incidences are unknown. The presentation of aplasia of the epiglottis can vary in nature and timing depending on other laryngeal abnormalities due to common embryological origin. Long‐term aspiration is a known cause of chronic pulmonary suppuration and the eventual development of bronchiectasis 3, 4. The case presented here is the first reported case of bronchiectasis as a likely consequence of early‐life aspiration events due to aplasia of the epiglottis.

## Case Report

A 2‐year‐old male was referred for investigation of recurrent lower respiratory tract infections. On history, he was a term baby who was admitted to the special care nursery at birth for 12 days for suspected sepsis. His mother had gestational diabetes under good control. He had a complex medical background with dysmorphic features (low set ears, clinodactyly, micrognathia, and multiple ear creases), multiple midline malformations (cleft soft palate, penoscrotal abnormality, multi‐cystic right testicle, Atrial Septal Defect (ASD) and Ventricular Septal Defect (VSD), everted eyelids), feeding difficulty, abnormal cry, hearing impairment and speech delay. Renal and cranial ultrasounds were normal. Neonatal screening was negative for cystic fibrosis. Neurological assessment at birth and subsequently was normal.

In the first 12 months of life, he had recurrent upper respiratory tract infections, and some of the episodes were associated with wheeze. He also had protracted episodes of wet cough with or without viral or febrile illness. There was no history suggestive of upper airway obstruction, but his cry was noted to be soft. He had feeding difficulty and failure to thrive with weight below the third centile. Feeding difficulty was attributed to the cleft palate. Both VSD and ASD had spontaneous closure in the first year of life. Cleft palate was repaired at 10 months of age without any major complications. Despite the repair, the child continued to have recurrent lower respiratory tract infections and chronic wet cough with bilateral crackles, even during periods of wellness. A chest X‐ray at the time of referral showed prominent bronchovascular markings with airspace change in the right middle lobe and left lower lobe. The wet cough persisted despite prolonged courses of oral antibiotics. He was thus further investigated for the cause of this chronic wet cough with a computed tomography (CT) chest, flexible bronchoscopy and immunological investigations.

**Figure 1:**
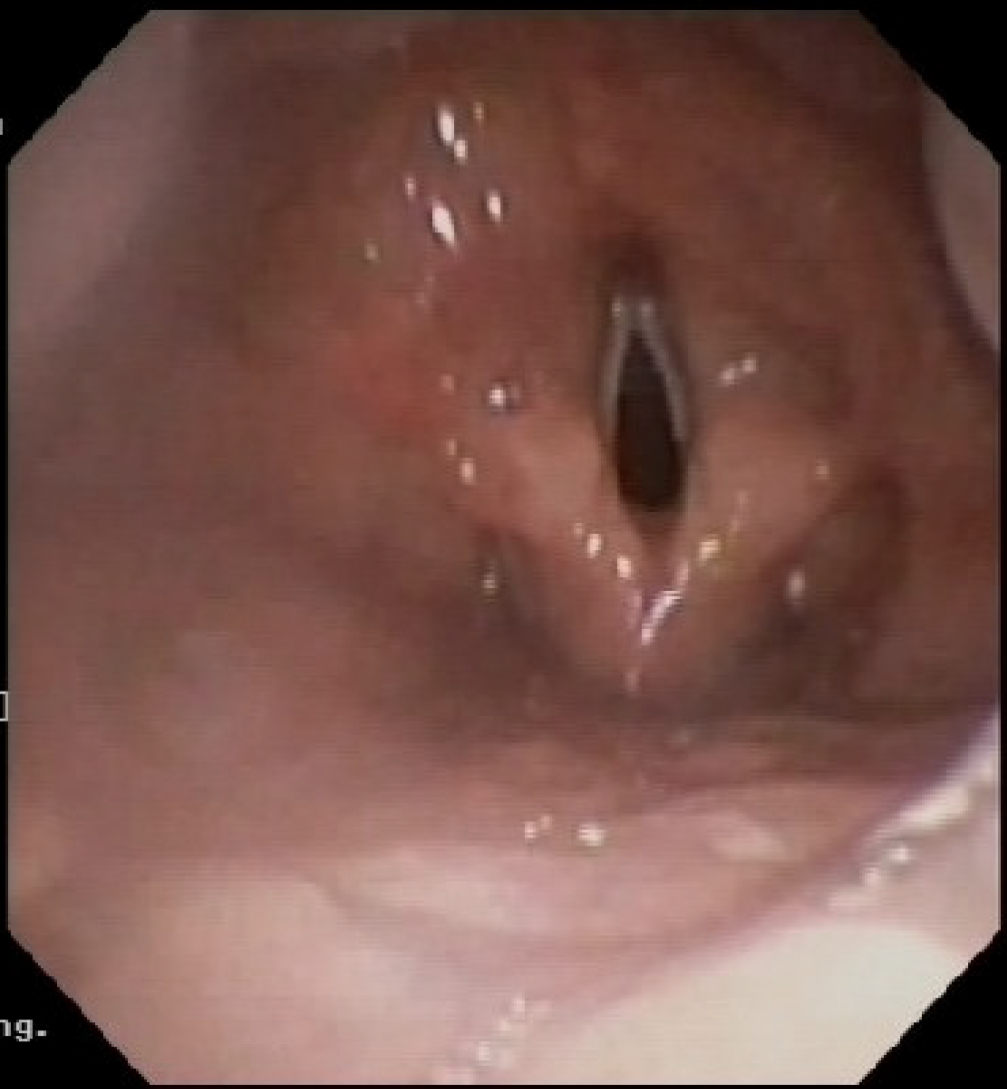
Laryngeal Introitus with Aplasia of epiglottis.

**Figure 2:**
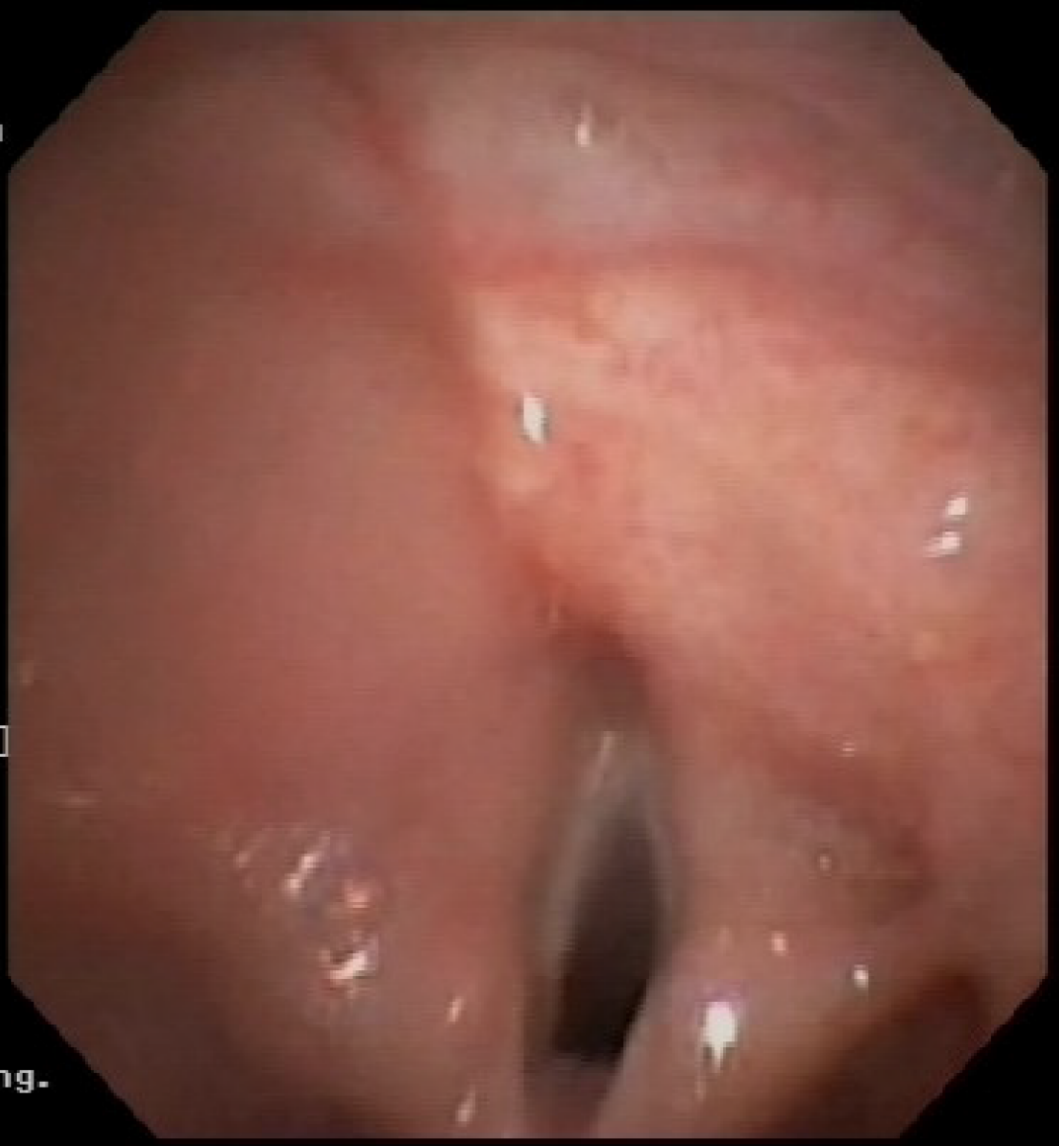
Laryngeal Introitus (Anterior View) showing absent epiglottis.

Immunological and aero‐allergen screening tests all demonstrated normal limits. Flexible bronchoscopy indicated the absence of an epiglottis. The appearance was consistent with agenesis or aplasia of epiglottis (Figs. 1, 2 and Video S1, Supporting Information). Other findings included widespread bronchitis, mucous plugging of right middle lobe, and branching anomalies. Vocal cord function was normal. Bronchoalveolar lavage fluid (BALF) showed neutrophilia (92% cells neutrophils) and cultured *Pseudomonas aeruginosa*, *Hemophilus influenzae* and *Streptococcus pneumoniae* in significant amounts. CT chest showed atelectasis in the right middle lobe with mild multi‐lobar bronchiectasis, most prominent in the lower lobes bilaterally. He was treated with intravenous anti‐Pseudomonal antibiotics and was started on oral azithromycin for 12 weeks. He was also started on airway clearance therapy with chest percussions. Video fluoroscopic swallow studies (VFSS) were performed where the child demonstrated prompt swallow triggers and adequate airway protection and was thus not recommended for a modified diet.

Currently, at 4 years of age, he is progressing well with resolution of his wet cough. He is maintained on prophylactic macrolides and chest physiotherapy and remains free of chronic cough and is regularly assessed by speech therapists and respiratory physicians. He continues to have mild speech delay but is fully orally fed and is otherwise neurologically normal. He has a small deficiency in the posterior part of the soft palate resulting in intermittent nasal regurgitation. He is observed to have early digital clubbing.

## Discussion

Congenital absence of the epiglottis is a rare condition. It is more commonly described in the literature as part of a syndrome, such as Nager syndrome, short polydactyly syndrome of the Majewski type, Pierre Robin sequence, or other non‐specific syndromic features 5. Isolated congenital aplasia of the epiglottis is less commonly described in the literature (Table 1).

**Table 1:** Summary of case reports on aplasia of epiglottis.

Study	Presentation	Interventions	Outcome
Constantinides and Cywes 6	6‐day‐old female presented with episodes of choking and cyanosis in the context of complete median cleft of the mandible and suspected Pierre Robin Sequence	Tracheostomy	Tracheostomy obstruction led to death few weeks later
Reyes et al. 2	3‐month‐old female presented with stridor and subsequent obstructive sleep apnoea with no aspiration on repeated swallow studies	No interventions	At 8 years of age, she was being advised for weight loss for OSA
Koempel and Hollinger 1	3‐month‐old female presented with work of breathing and stridor	Fundoplication and gastrostomy feeds	At 17 months, she was not attempting vocalization or swallowing
Bonilla et al. 7	Newborn with stridor, work of breathing and aspiration pneumonitis on a background of dysmorphic features	Tracheostomy	Successful decannulation at 7 years of age
Dritsoula and Thevasagayam 8	Newborn with retrognathia and presented with stridor. Issues with initiation and propagation of swallow were demonstrated on swallow studies	Fundoplication and PEG insertion at 7 weeks. Glycopyrrolate for secretions	Well at 11 months but continued total gastrostomy feeds
Tay et al. 5	3‐year‐old female with abnormal swallow test on the background of Nager syndrome	Tracheostomy and nasogastric feeds	Not detailed
Tay et al. 5	18‐month‐old female with VFSS demonstrated aspiration on the background of Nager syndrome	PEG feeds	Not detailed
Shahi and Singh 9	Incidental finding in 30‐year‐old female with no symptomology at elective procedure	None	Not detailed
Kim et al. 10	Incidental finding in 33‐year‐old female presenting with acute tonsillitis	None	Acute tonsillitis resolved. Observed to have compensatory action by hypertrophied lingual tonsil on video fluoroscopic barium swallow test
Prasad 11	Six cases of babies 1–3 days old with acute severe respiratory distress, cyanosis and bradycardia in the context of isolated agenesis of epiglottis	Supraglottic closure with temporary tracheostomy and NG feeding. Glottis restored 3–6 months postoperatively when satisfied with pharyngoesophageal coordination	Five of six cases well and thriving at the 6‐month follow up. One case passed before intervention could take place
Kilinc et al. 12	10‐month‐old male with Pierre Robin Sequence presenting with aspiration pneumonia, weight loss, and subsequent demonstrated impaired swallow	Nasogastric feeding and swallow rehabilitation programme	Improved swallow at 12 months with no further respiratory or weight issues

Development of the epiglottis begins around day 33 of life and is complete by day 48 of life, concurrent with the development of limbs and digits. Disruptions to organogenesis during this period result in abnormalities found in oro‐faciodigital syndromes in addition to abnormalities of the epiglottis 1, 5.

The condition may be an incidental finding or it may present as aspiration events with associated pneumonitis, recurrent respiratory tract infection, or upper airways obstruction. Most cases of aplasia of the epiglottis in the literature have been managed with tracheostomy and feeding interventions. Our child certainly had significant feeding difficulty early in life, which was attributed to his cleft palate; it is unclear if the absence of epiglottis contributed to this feeding and swallowing difficulty. It is likely that the high frequency of lower respiratory tract infections despite the palatal surgery were consequent to persistent aspiration due to an absent epiglottis.

Recurrent aspiration secondary to structural causes, such as trachea–oesophageal fistula, or neurological impairment, such as cerebral palsy, are known reported causes of recurrent lower respiratory tract infections and bronchiectasis in children 13. To our knowledge, bronchiectasis has never been reported with absent epiglottis (Table 1).

The role of the epiglottis in airway protection is unclear. Surgical epiglottectomy in adults has not been associated with swallowing difficulty or increased aspiration risk 14. However, swallowing impairment and aspiration are present in many cases of absent epiglottis described here, particularly at a young age 5, 6, 7, 8. Whether its link to bronchiectasis in our case was causal remains conjectural as we did not find any other cause for his underlying bronchiectasis. It is also postulated that compensatory airway‐protective mechanisms may be acquired with growth in otherwise neurologically intact children with normal vocal cord function, even in the absence of epiglottis. This may explain the absence of significant aspiration in his swallow study at 3 years of age. This would also be supported by the absence of swallowing impairment in the two adult cases of congenital epiglottic aplasia. Kilinc 12 describes an objective improvement in aspiration, with swallow rehabilitation further demonstrating learned compensation in the absence of epiglottis. Regardless, aspiration prevention measures have been undertaken in a majority of the cases described in the literature in the management of these patients. In the absence of these measures, as in our case, the first presentation of aplasia of the epiglottis may be the sequelae of aspiration, such as recurrent respiratory tract infections and bronchiectasis.

### Disclosure Statement

Appropriate written informed consent was obtained for publication of this case report and accompanying images.

## Supporting information


**Video S1.** Bronchoscopic video of aplasia of the epiglottis.
